# Computational analysis reveals temperature-induced stabilization of FAST-PETase

**DOI:** 10.1016/j.csbj.2025.03.006

**Published:** 2025-03-05

**Authors:** Peter Stockinger, Cornel Niederhauser, Sebastien Farnaud, Rebecca Buller

**Affiliations:** a Research Centre for Health & Life Sciences, Coventry University, Coventry CV1 5FB, United Kingdom; b Competence Center for Biocatalysis, Zurich University of Applied Sciences, Einsiedlerstrasse 31, Wädenswil 8820, Switzerland

**Keywords:** Plastics degradation, Molecular dynamics, PET, Thermostability, Biocatalysis, Enzyme Engineering, Machine learning, PETase

## Abstract

More than 10 % of global solid waste consists of poly(ethyleneterephthalate) (PET). Among other techniques, PET hydrolases (PETases) can be used to depolymerize this plastic. However, wildtype PETases exhibit poor specific activities and insufficient thermostability, limiting their use in depolymerization processes which require high temperatures. In 2022, machine learning-aided enzyme engineering of a PETase stemming from the bacterium *Ideonella sakaiensis* (*Is*PETase) resulted in a more functional, active, stable, and tolerant variant (FAST-PETase). To rationalize the molecular basis of FAST-PETase’s improved thermal stability, we performed comparative Constraint Network Analysis (CNAnalysis) and Molecular Dynamics (MD) simulations of wildtype *Is*PETase (WT-PETase) and FAST-PETase at 30°C and 50°C identifying thermolabile sequence stretches in the wildtype enzyme. Further analysis of the backbone flexibility revealed that all mutations of FAST-PETase affected these critical regions. Counterintuitively, the *in-silico* analyses additionally highlighted that the flexibility of these regions decreased at 50°C in FAST-PETase, instead of exhibiting increased flexibility at higher temperature as would be expected from thermodynamic considerations. This effect was confirmed by physical energy calculations, which suggest that temperature-dependent conformational changes of FAST-PETase decrease the free energy of unfolding (ΔG(stability)) and rigidify the enzyme at elevated temperatures enhancing stability. Looking forward, these findings might help guide the rational engineering of protein thermostability and contribute to our understanding of the thermal adaptation of thermophilic enzymes.

## Graphical Abstract

**Figure:**
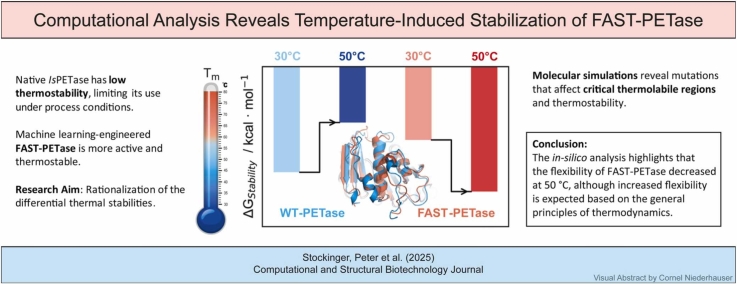

## 1 Introduction

To achieve a circular economy, the ability to recycle the growing amount of plastic waste is considered to be of high priority. [1] With PET accounting for around 12 % of the global solid waste volume (8 % by weight), the development of efficient and economical de- and repolymerization processes is therefore key. [2], [3] One such PET depolymerization strategy is the plastics’ enzymatic hydrolysis, first reported in 2005. [4], [5] Although several enzymes with PET-hydrolyzing activities have been identified, such as esterase, lipase and cutinase enzyme annotations, [6], [7], [8], [9] expensive PET pretreatments are necessary to achieve acceptable depolymerizing activities with these promiscuous enzymes. [10], [11], [12] In 2016, PETase from *Ideonella sakaiensis* (*Is*PETase) was reported to be suitable for the operation at temperatures between 30 and 50 °C. [13] However, this enzyme was found to be poorly stable, with loss of activities after 24 hours incubation at 37 °C. [14] Since PET becomes more accessible near its glass transition temperature (65–75 °C), [15] increasing T_m_ and optimal operating temperature of *Is*PETase is desirable. Thus, several enzyme engineering strategies have been applied to optimize *Is*PETase’s utility by enhancing its thermostability, activity and process conditions. [14], [16], [17], [18], [19] As a result, two stabilized variants, ThermoPETase and DuraPETase, were generated, which displayed higher thermostability (ΔT_m_ 10 and 30 °C, respectively) and activity at elevated temperatures (> 60 °C) but lower activity when mild temperatures (30–50 °C) were applied in depolymerization experiments. [14], [16], [20] Building on initial engineering attempts, *Is*PETase was further optimized by harnessing a structure-based machine learning algorithm, [14] which was combined with combinatorial mutagenesis. This strategy resulted in the **f**unctional, **a**ctive, **s**table and **t**olerant *Is*PETase variant (FAST-PETase) with optimized PET-hydrolytic activity compared to wildtype *Is*PETase, and the previously most active variants ThermoPETase and DuraPETase (between 30 and 50 °C). [20]

Recent studies utilizing MD simulations and QM/MM calculations on FAST-PETase and PETase with and without a PET substrate model suggest that the enhanced catalytic efficiency of FAST-PETase in PET degradation is primarily due to two factors: control of substrate conformation and transition state stabilization. [21], [22] Control of substrate conformation is achieved by the enzyme’s ability to induce reactive conformations of the PET substrate, which are not found when the substrate is free in solution. This pre-organization at the active site, particularly at 50°C, optimizes substrate alignment in FAST-PETase’s active site for catalysis. [21] Furthermore, transition state stabilization by FAST-PETase seems to be enhanced through an N233K mutation, which locally alters the depolymerases’ structure increasing the basicity of Asp206 and strengthening the interaction with His237. This interaction reduces the free energy barrier of the acylation stage (12.1 kcal/mol for FAST-PETase vs. 16.5 kcal/mol for wildtype *Is*PETase), ultimately accelerating the reaction rate. [22]

In addition to the improved transition state stabilization and the fine-tuned substrate binding conformation at 50 °C, FAST-PETase exhibits a notably improved thermal stability, with a T_m_ of 67 °C – an increase of 18 °C compared to WT-PETase’s T_m_ of 49 °C. Interestingly, unlike other depolymerases, FAST-PETase performs best at 50°C, rather than at temperatures near the glass transition of PET (65–75 °C). At 50°C, FAST-PETase releases more than twice the amount of PET monomers (defined as the sum of terephthalic acid (TPA) and mono(2-hydroxyethyl)terephthalate (MHET)) in 24 hours compared to ThermoPETase and DuraPETase. This improved performance at a specific temperature suggests thermal adaptation of the biocatalyst, e.g., that the enzyme’s dynamic properties have been fine-tuned for efficient catalysis under specific conditions. [23], [24] In contrast, ThermoPETase and DuraPETase seem to rely on structural rigidity to maintain their function at higher temperatures, prioritizing stability over dynamic flexibility.

While hydrogen-bonding analysis of WT- and FAST-PETase crystal structures indicate stabilizing effects of the introduced mutations (N233K/R224Q/S121E/D186H/R280A), [20] the influence of the mutations on protein dynamics is less well understood. Thus, we hypothesized that a more detailed analysis of the underlying protein dynamics would provide insights into the molecular basis of FAST-PETase’s thermal adaptation whilst also enabling identification of novel hotspot positions for future enzyme engineering campaigns. In support of our hypothesis, Creveld et al. reported the ability to differentiate flexible regions which promote unfolding from those with catalytic importance by performing MD simulation of cutinase from *Fusarium solani pisi*. Regions prone to unfolding were identified and proposed to serve as hotspot areas for mutations. [25] Targeted mutagenesis of equivalent regions in the related *Glomerella cingulate* cutinase was later shown to improve thermostability without influencing enzyme activity. [26] As the discussed cutinases are part of α/β-hydrolase family (which comprises all currently known PET-degrading enzymes), [27] following a similar approach to study unfolding regions in PETase seemed promising.

Here, structural dynamics of WT- and FAST-PETase were studied by performing both, CNAnalysis thermal unfolding simulations and MD simulations at 30 and 50 °C. The simulations indicated a temperature-induced stabilization of FAST-PETase, which was confirmed by secondary-structure analysis and free energy calculations.

## 2 Results

### 2.1 Constraint Network Analysis (CNAnalysis)

Constraint Network Analysis (CNAnalysis) can be used to identify variations with regards to rigidity and flexibility at optimal temperatures of homologous mesophilic and thermophilic proteins. [28] Based on graph theory, global and local flexibility/rigidity characteristics of proteins can be derived from single or multiple protein structures to support data-driven protein engineering with the aim to improve the proteins’ thermal stability. [29]

As a first step to analyze flexibility/rigidity characteristics of the selected PETase variants, thermal unfolding simulations of a single constraint network derived from crystal structures of WT-PETase and FAST-PETase (PDB accessions 5XJH and 7SH6, respectively) were performed utilizing the CNAnalysis Web Interface (http://www.cnanalysis.de). [30] A constraint network refers to a model for which the protein's atomic or residue-level structure is represented as a network of nodes (atoms or residues) connected by edges, which correspond to stabilizing interactions such as hydrogen bonds, salt bridges, hydrophobic interactions, and covalent bonds. Through thermal unfolding simulations, a stability map can be derived from the constraint network, which enables the visualization of how rigid contacts are distributed and how they are impacted by thermal stress, providing insights into the protein’s structural stability.

**Fig. 1:**
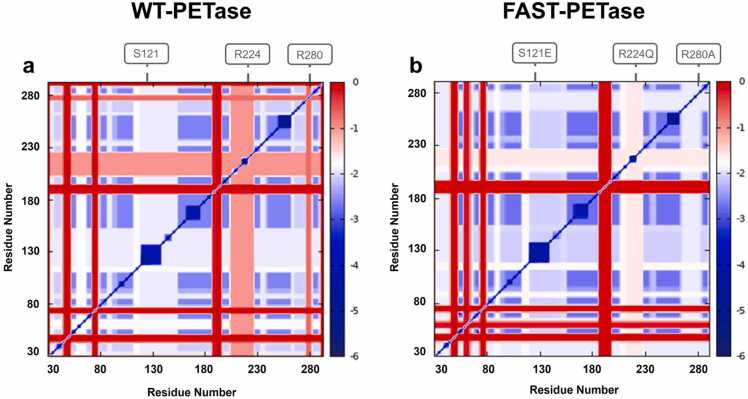
CNAnalysis Stability Map. Stability map of (**a**) WT-PETase and (**b**) FAST-PETase resulting from thermal unfolding simulations of a single network derived from the corresponding crystal structures (PDB accessions 5XJH and 7SH6, respectively). The red color indicates pairs of residues which form weak rigid contacts, while the intensity of the blue color correlates to increasingly stronger rigid contacts.

When analyzing the stability maps of the PETase variants (Fig. 1), it became evident that while rigid contacts in sequence region 55–60 of FAST-PETase were weakened compared to WT-PETase, all other differentiating regions exhibited stronger rigid contacts in FAST-PETase. For instance, rigidity scores of contacts within region 110–155 around position 121 (-1.8 vs −2.2) and region 200–230 around position 224 (-1.0 vs −1.5) increased for almost all contacts. Interestingly, the region surrounding key mutation R280A in FAST-PETase, which alters the substrate binding site conformation to allow stronger substrate interactions, [18] also showed more rigid contacts (-0.8 vs −2.0) not seen around R180 in WT-PETase.

To further investigate the origin of thermal instability in WT- and FAST-PETase, unfolding nuclei frequencies were calculated using thermal unfolding simulations on an ensemble of networks (50 network topologies). Unfolding nuclei are weak spots within the protein, which can be considered as starting points of the thermal unfolding processes. Residues with higher unfolding nuclei frequencies represent weaker spots of the protein. Through the prediction of unfolding nuclei, at least four potential hotspots for further stability engineering of WT- or FAST-PETase toward increased thermal adaptation were identified around residues Y69, A82, V107 and M156 (Supplementary Fig. S1). Regions around substitutions S121E and D186H in FAST-PETase displayed a considerable drop in the unfolding nuclei frequencies compared to the corresponding WT-PETase frequencies. Calculating the difference between WT- and FAST-PETase frequencies mainly resulted in positive values indicating the overall reduction of unfolding nuclei.

### 2.2 Molecular Dynamics (MD) simulations

While CNAnalysis's thermal unfolding simulations offered a straightforward way to pinpoint protein regions influencing the rigidity and flexibility of WT- and FAST-PETase, molecular insights into residue interactions and conformational changes cannot be identified by this methodology. To confirm whether protein dynamics influences the PETase variants’ thermal stability, we opted to carry out MD simulations on both, the WT- and FAST-PETase, at 30°C and 50°C.

**Fig. 2:**
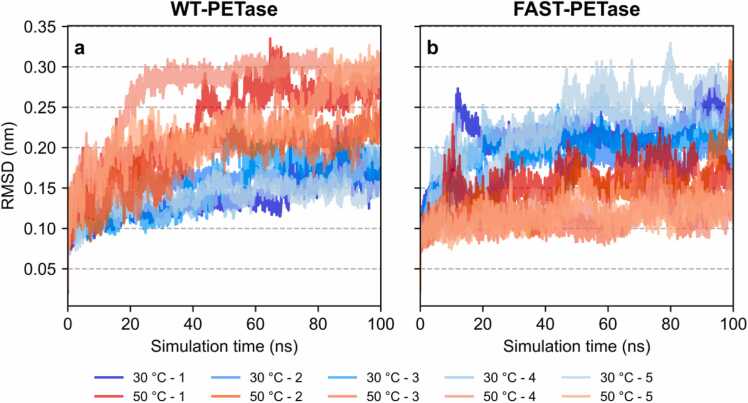
MD Simulation-derived RMSD analysis. RMSD was calculated for all MD simulation replicates of (**a**) WT- and (**b**) FAST-PETase. The 30 °C simulations are displayed using blue colors while the 50 °C simulations are displayed in shades of red. To reduce the noise in the original data (Supplementary Fig. S2), Savitzky-Golay filter (window size: 11, polynomial order: 3) was applied. The original data and scripts used for plotting have been uploaded to https://github.com/ccbiozhaw/FAST-PETase_stability.

After extensive three-step equilibration (constant volume and temperature (NVT) with restraints, constant pressure and temperature (NPT) with restraints, and free constant pressure and temperature (NPT)), five separate production runs were simulated for 100 nanoseconds each. Subsequently, root mean square deviation (RMSD) of the protein backbone molecules was analyzed and visualized for all simulation systems (Fig. 2). For WT-PETase, all replicates of the 30 °C simulation system displayed RMSD values converging between 0.125 and 0.2 nm. RMSD values of all replicates simulated at 50 °C converged in a range between 0.175 and 0.325 nm. Unexpectedly, RMSD analysis of FAST-PETase revealed an unusual behavior of protein flexibility: While RMSD values ranging between 0.175 and 0.3 nm were found for all replicates of the 30 °C simulation system, the RMSD values of all replicates simulated at 50 °C converged in a lower range between 0.1 and 0.15 nm. This inverted behavior is also reflected in the radius of gyration (Rg) and solvent accessible surface area (SASA). While SASA and Rg of WT-PETase were found to be higher at 50°C than at 30°C, FAST-PETase consistently displayed decreased SASA and Rg at 50°C (Supplementary Fig. S3 and S4).

**Fig. 3:**
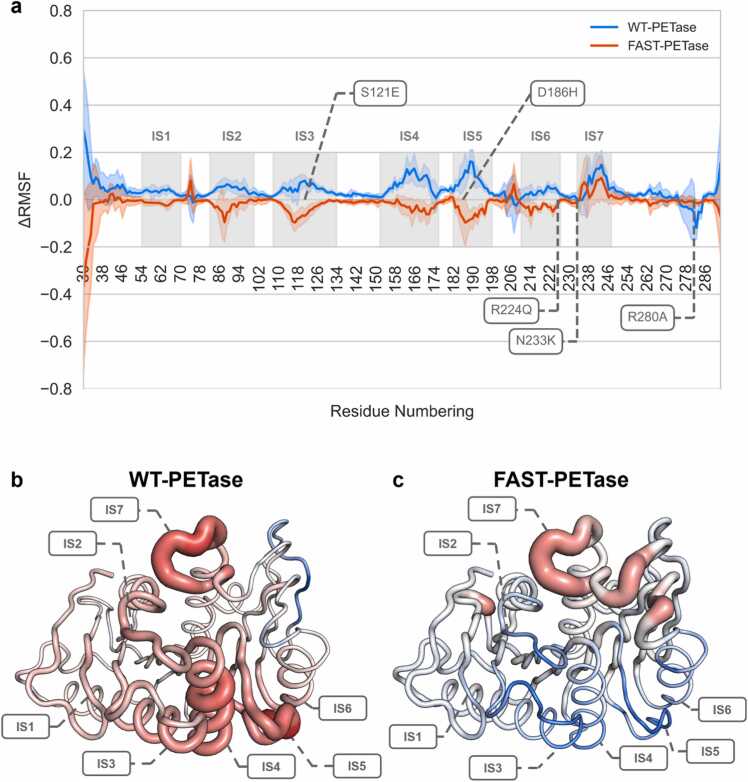
MD simulation-derived RMSF analysis. RMSF was calculated for all simulation replicates of the 30 and 50 °C simulations of WT- and FAST-PETase. To assess this parameter’s impact on thermostability, ΔRMSF values were calculated by subtracting the mean RMSF value of all 30 °C replicates from the mean RMSF value of all 50 °C replicates ($\Delta \mathrm{RMSF}={\mathrm{mean}(\mathrm{RMSF}}_{50{}^{\circ}C})-\mathrm{mean}({\mathrm{RMSF}}_{30{}^{\circ}C})$). (**a**) ΔRMSF of WT- and FAST-PETase are shown in blue and red, respectively. Depicted error bars illustrate the spread of the replicates. Derived instability regions, named IS1-IS7, are highlighted with grey boxes. Mutations distinguishing WT- and FAST-PETase are marked using dashed lines and boxes. The B-fa**c**tor entries for (**b**) WT- (PDB: 5XJH) and (**c**) FAST-PETase (PDB: 7SH6) were replaced with calculated ΔRMSF values to visualize the stabilized regions. Using PyMOL, these values were displayed as a range from −0.2–0.2, where positive values are highlighted in red with thick ribbons and negative values in blue with thin ribbons. For clarity of visualization, ΔRMSF values corresponding to the terminal regions (residues 30–50 and 290–312) were excluded.

Analysis of backbone root mean square fluctuation (RMSF) further confirmed these results, identifying several distinct instability regions (IS) for which WT-PETase exhibited significantly higher RMSF values during the 50 °C simulation replicates compared to the simulations carried out at 30°C (IS1–IS6, Fig. 3). In contrast, FAST-PETase showed higher RMSF values at 30°C and lower RMSF values at 50°C. This opposite behavior becomes evident when comparing the ΔRMSF betwen 30 an 50°C for both enzymes. In regions IS1-IS6, WT-PETase exhibited positive ΔRMSF values, indicateing temparture-induced unfolding, whereas the negative ΔRMSF in these regions for FAST-PEtase suggest increased rigidity at higher temperatures. However, this trend did not apply to instability region seven (IS7), which displayed only a slightly reduced ΔRMSF compared to the WT. Notably, FAST-PETase mutations S121E, D186H, R224Q and N233K are located within or are adjacent to these instability regions (Fig. 3).

To further investigate differences in the structures of the 30 and 50 °C PETase simulation ensembles, secondary structures of selected conformations (frames at 0, 10, 20, 30, 40, 50, 60, 70, 80, 90 and 100 ns) were analyzed for all replicates of WT- and FAST-PETase. At higher temperatures, WT-PETase replicates displayed a decrease of α-helices and loops/disordered regions while the content of 3–10 helices and extended strands increased. By contrast, FAST-PETase displayed the opposite behavior forming loops and helices at higher temperatures while the content of 3–10 helices and extended strands was decreased in comparison to the 30 °C ensembles (Supplementary Fig. S5). This finding was further supported by a quantitative comparison of the average α-helix and 3–10 helix content in the selected conformations at 50 °C: In comparison to WT-PETase, FAST-PETase exhibited an increased α-helix content (+2.1 %; p = 0.0042) and a decreased 3–10 helix content (-1.8 %; p = 0.0097).

**Fig. 4:**
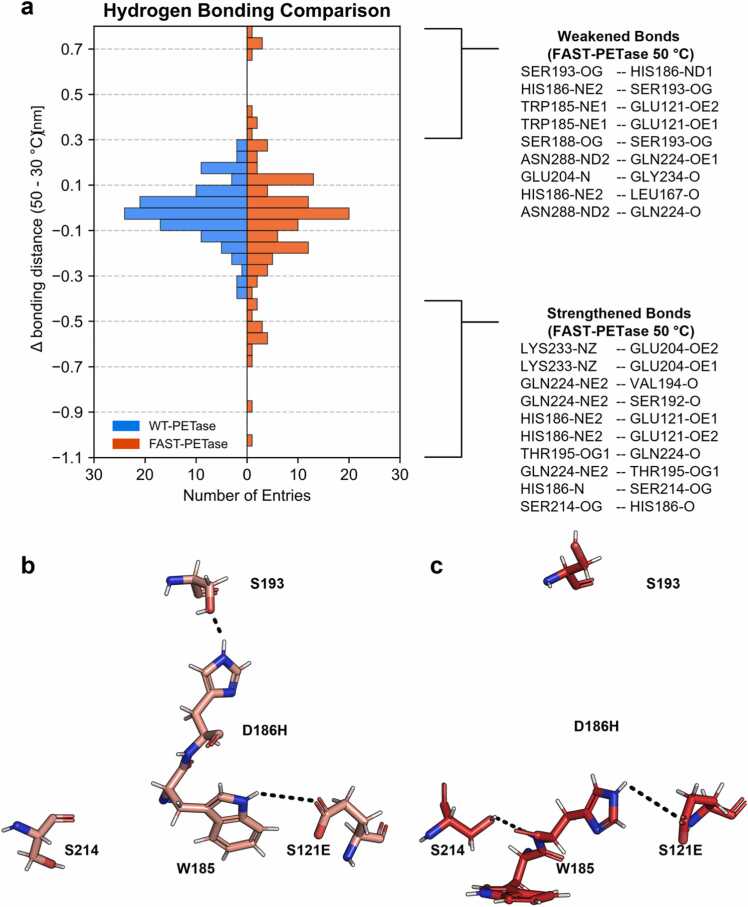
MD Simulation-based Hydrogen Bonding Analysis. (a) Mirrored histogram (bar width = 0.05 nm) highlighting hydrogen bonds that are more frequently formed or broken in WT- (blue) and FAST-PETase (orange) upon increased simulation temperature (30 vs 50 °C). Atom names correspond to those specified in the PDB files. Temperature was found to have a stronger impact on the hydrogen bond distances of FAST-PETase residues compared to those of WT-PETase as shown by the broader distribution of the bonding distance deltas (Δ), calculated by subtracting the average bonding distance at 30 °C from the 50 °C values. This rearrangement is further illustrated by the hydrogen-bonding network of residues S121E, W185, D186H, S193, and S214 in FAST-PETase, simulated at (**b**) 30 °C and (**c**) 50 °C.

Prior studies hypothesized that additional hydrogen bonding and salt bridge interactions contribute to the improved thermal stability of FAST-PETase. [20] In detail, interactions of S121(E), D186(H), R224(Q) and N233(K) with residues N172, S192, S193, E204 and N233(K) were identified as relevant for the improved stability. Thus, hydrogen bonding analysis of these nine residues at 30 and 50 °C was performed using all WT- and FAST-PETase simulations trajectories. Changes of the mean bond distances between all identified hydrogen bonding atom pairs were evaluated by calculating the differences between the 30 and 50 °C simulation systems (Fig. 4, Table 1). Overall, hydrogen bond distances in FAST-PETase were found to exhibit stronger changes in dependence of temperature (Δ bonding distance (50°C – 30°C) ranging from −1.022 to 0.766 nm) than those in WT-PETase (Δ bonding distance (50°C – 30°C) ranging from −0.381 to 0.257 nm).

**Table 1:** MD simulation-based hydrogen bonding analysis of FAST-PETase.

Hydrogen Bond	Mean Distance [nm]		Δ [nm]
	30°C simulations	50°C simulations	
SER193-OG -- HIS186-ND1	0.525	1.292	0.766
HIS186-NE2 -- SER193-OG	0.411	1.161	0.750
TRP185-NE1 -- GLU121-OE2	0.715	1.451	0.736
TRP185-NE1 -- GLU121-OE1	0.717	1.451	0.734
SER188-OG -- SER193-OG	0.417	1.073	0.656
ASN288-ND2 -- GLN224-OE1	0.547	0.974	0.427
GLU204-N -- GLY234-O	0.314	0.713	0.398
HIS186-NE2 -- LEU167-O	0.503	0.859	0.356
ASN288-ND2 -- GLN224-O	0.355	0.680	0.325
LYS233-NZ -- GLU204-OE2	1.036	0.636	−0.400
LYS233-NZ -- GLU204-OE1	1.036	0.632	−0.404
GLN224-NE2 -- VAL194-O	1.130	0.642	−0.488
GLN224-NE2 -- SER192-O	0.990	0.482	−0.508
HIS186-NE2 -- GLU121-OE1	1.178	0.657	−0.521
HIS186-NE2 -- GLU121-OE2	1.190	0.658	−0.532
THR195-OG1 -- GLN224-O	1.158	0.599	−0.559
GLN224-NE2 -- THR195-OG1	1.449	0.787	−0.662
HIS186-N -- SER214-OG	1.258	0.386	−0.872
SER214-OG -- HIS186-O	1.395	0.373	−1.022

Hydrogen bonds involving the residues E121, H186, N172, S192, S193, E204, Q224, and K233 were analyzed and ranked by the difference (Δ) in their mean distances between 30 °C and 50 °C. Atom names (e.g. OG, ND1 or NE1) correspond to those specified in the PDB files. A negative Δ value indicates that the hydrogen bond is stronger at higher temperatures, while a positive Δ value indicates weakening. This table presents a selection of bonds from the full dataset (Table S2), focusing on those bonds that displayed average distances below 0.8 nm. Although these distances may appear quite large for hydrogen bonds (typically around 0.35 nm), it is important to note that hydrogen bonds break and form dynamically over the simulation trajectory. The hydrogen bonding detection algorithm also considers bonds that are only rarely formed.

**Fig. 5:**
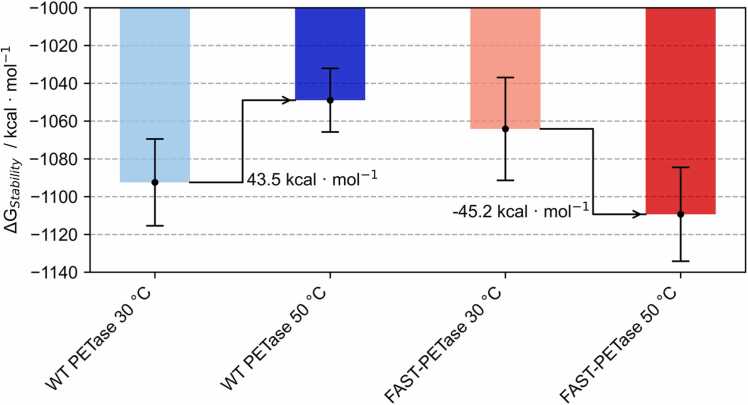
MD Simulation-based Physical Energy Calculations. Depiction of the average EvoEF2 ΔG(stability) of the five end frames from the MD simulations of WT- and FAST-PETase (blue and red shades, respectively). The lighter colors correspond to calculation based on the MD simulations at 30°C and the darker colors show the values derived from the MD simulations at 50°C. The error bars show the standard deviation of the five end frames.

To further support our hypothesis of a temperature-induced stabilization of FAST-PETase, we calculated the free energy of unfolding (ΔG(Stability)) using a set of physical energy functions (EvoEF1 [31], EvoEF2 [32] and FoldX [33]) by applying them on the last state of all simulation replicates (Fig. 5 & Supplementary Fig. S6). Consistently, all methods captured a decreased stability of WT-PETase at higher temperature, while FAST-PETase was found to be stabilized at 50°C (Supplementary Table S3).

## 3 Discussion

FAST-PETase, obtained through ML-assisted engineering, exhibits a temperature-dependent dynamic behavior which is distinct from its parental enzyme. In this context, stability map analysis revealed that the strength of rigid contacts is increased in FAST-PETase compared to the wildtype enzyme, while MD simulations identified instability regions (IS1-IS7) which display a different behavior as a function of the applied temperature and the investigated variant. WT-PETase displayed significantly higher RMSF values in instability regions at 50°C compared to 30°C, indicative of increased flexibility at higher temperatures. Surprisingly, however, in FAST-PETase instability regions IS1, IS2, IS3, IS4, IS5, and IS6 exhibited decreased flexibility at higher temperatures, suggesting a temperature-induced stabilizing effect. Importantly, in this context, many mutations present in FAST-PETase are within or adjacent to the instability regions, suggesting a role in modulating the protein's stability and dynamics through the reorganization of hydrogen bonding patterns potentially causing the thermal adaptation. For example, key mutations S121E and D186H impact the hydrogen bonds between residues E121 and W185, as well as H186 and S193, while the collapse of these bonds at 50 °C is compensated, *inter alia*, by the formation of stabilizing bonds between E121 and H186, as well as H186 and S214.

The putative temperature-induced stabilization of FAST-PETase is further supported by a secondary structure analysis: While WT-PETase displayed a lower proportion of α-helices and a higher proportion of 3–10 helices at 50 °C, FAST-PETase maintains its α-helix content even at elevated temperature. Notably, 3–10 helices are less abundant in nature and constitute only 10–15 % of all helices in protein secondary structures. This type of helix is stabilized by intramolecular hydrogen bonds between the carbonyl group of amino acid n and the amide group of the amino acid n + 3. However, the formation of van der Waals contacts and hydrogen bonds is less favorable in a 3–10 helix and in general, the α-helix is considered to be more stable. Notably, 3–10 helices are typically observed as extensions of α-helices suggesting that the 3–10 helix may serve as an intermediary conformation potentially playing a role in the initiation of α-helix folding. [34] Elevated temperatures are known to induce such conformational changes in proteins, altering their secondary structure elements. Thus, the observed structural differences between 30°C and 50°C likely reflect the varying degrees of structural adaptation of the PETase variants to thermal stress. [35], [36]

The structural characteristics of a thermophilic enzyme at higher temperatures often resemble those of a mesophilic enzyme at lower temperatures. [23], [24] For instance, MD simulations of adenylate kinases have shown similar local mobilities, folding free energy, and enzymatic activities in kinases from mesophilic and hyperthermophilic origins at their optimal temperatures. In this context, it has been proposed that this similarity arises from a shared protein energy landscape within their optimal temperature environments. [37], [38] Moreover, experimental evidence shows that temperature does not only affect enzyme stability, but also the distribution of active enzyme conformations. [35] Currently, however, computational enzyme design cannot yet mimic Nature’s temperature-dependent conformational finetuning which might explain typical characteristics of thermostabilized enzymes: A) high stability yet poor activity at high temperatures (e.g. PROSS5-PETase [39]) or B) high activity and stability at elevated temperature yet poor activity at ambient temperatures (e.g. ThermoPETase and DuraPETase) [14], [16], [20], [40]. In contrast to other engineered PETases, FAST-PETase does not exhibit this trade-off, as its stabilization is only induced at elevated temperatures, allowing it to maintain both stability and activity across a broader temperature range. Notably, HOT-PETase, developed through directed evolution, has been reported to exhibit activity across an even wider temperature range than FAST-PETase and demonstrates best-in-class thermal stability, with a T_m_ of 82.5 °C. Similar to FAST-PETase, mutations in HOT-PETase appear to enhance structural stability while maintaining the flexibility of the active site, a combination rarely seen in rigorously thermostabilized variants. [41]

Looking forward, the design of thermostable and active *Is*PETase variants could be further guided by identifying naturally evolved thermophilic proteins and adapting their binding sites to fit the desired substrate through directed evolution. [42], [43] Harnessing these types of enzymes as starting scaffolds has proven exceptionally successful with thermostable cutinase variants (LCC^ICCG^ and PES-H1^L92F/Q94Y^) [44], [45] significantly outperforming both FAST-PETase and HOT-PETase under process-like conditions. [46] By learning from these templates, the *in-silico* creation of temperature-adapted enzymes might soon become a reality.

## 4 Conclusion

Our comparative MD-driven study of WT- and FAST-PETase sheds light on the thermal adaptation of proteins, specifically highlighting the role of flexibility regions and temperature-dependent conformational changes that impact both stability and functionality. We anticipate that analyzing conformational enzyme ensembles with energy function will further improve the accuracy of thermal stability predictions offering potential for *in-silico* pre-screening of enzyme variants. Overall, this work not only supports the rational design of thermostable enzymes but also contributes to a broader understanding of thermal adaptation in thermophilic enzymes.

## 5 Methods

### 5.1 CNAnalysis

Thermal unfolding simulations on an ensemble of networks derived from crystal structures of WT-PETase and FAST-PETase (PDB accessions 5XJH and 7SH6, respectively) were performed utilizing the CNAnalysis Web Interface (http://www.cnanalysis.de). [30] Overall, 50 network topologies were used for thermal unfolding simulations. Hydrophobic tethers were selected to stay constant during the simulations. Default E-cutoffs with initial value −0.1, terminal value −6.0 and stepsize 0.1 were selected. The stability map was generated analogously but was derived from a thermal unfolding simulation of a single network.

### 5.2 MD simulations

For both variants (WT- and FAST-PETase), side chain pKa values were calculated using PROPKA and PDB2PQR server (version 2.0.0). [47], [48] According to the results provided for a pH 8 and the parse forcefield, the residues’ protonation states were adapted using OpenMM Modeller class. The simulations were performed using OpenMM 7.4.125, on NVIDIA’s GPU computing platform CUDA. [49], [50], [51] General Amber force field (GAFF) was used with Amber14. [52], [53] Box padding of 1 nm was used with a cubic box which was solvated with water (tip4p-Ew water model). [54] Protein charge was neutralized, and an ionic strength of 0.1 M NaCl was applied. Energy was minimized until 10 kJ/mole tolerance energy. Two simulation systems were set up for both variants utilizing a pH 8 with temperatures of 303.15 K and 323.15 K, respectively. Langevin integrator was applied, [55] whereby a friction coefficient of 1/ps and a step size of 2 fs were utilized. Further, long range Coulomb interactions were computed using the Particle Mesh Ewald method with a 1 nm nonbonded cut-off for direct space interactions. During equilibration, a force of 100 kJ/mole* Å^2^ was applied to restrain the protein backbone. During solvent equilibration, the systems were simulated for 5 ns with NVT ensemble. Subsequently, Monte Carlo barostat and pressure coupling of 1 atm were applied and the restrained system was simulated for another 5 ns. Finally, the restraints were removed, and 5 ns of free equilibration were performed. With the resulting system, a production of 5 replicates à 100 ns was performed under periodic boundary conditions. After every 1000 steps, trajectories were saved.

### 5.3 RMSD and RMSF analysis

To compare the variants’ flexibility at different tempered systems, MDTraj was utilized to analyze RMSD (Fig. 2) and RMSF for all replicates. [56] In the case of RMSF analysis, the average of all replicates was calculated. The resulting average RMSF values of the 30°C system was subtracted from the according average RMSF values of the 50°C system to derive ΔRMSF (Eq. 1).

(1) $$\Delta \mathrm{RMSF}={\mathrm{mean}(\mathrm{RMSF}}_{50{}^{\circ}C})-\mathrm{mean}({\mathrm{RMSF}}_{30{}^{\circ}C})\;$$

Comparing ΔRMSF courses of WT-PETase and FAST-PETase enabled the identification of regions for which a) the largest impact of temperature and b) the largest difference between the variants was observed (Fig. 3).

### 5.4 SASA and Rg analysis

To compare the variants’ solvent accessible surface area (SASA) and radius of gyration (Rg) at different tempered systems, MDTraj was utilized to analyze every 100th frame of all replicates. [56] The utilized python scripts and output files were uploaded to the GitHub repository (https://github.com/ccbiozhaw/FAST-PETase_stability).

### 5.5 Secondary structure analysis

Dynamic alterations in secondary structure were observed by analyzing selected frames (at 0, 10, 20, 30, 40, 50, 60, 70, 80, 90 and 100 ns) via MDTraj’s implementation of the dictionary of protein secondary structure. [56], [57] The average occurrence of the assigned secondary structures were calculated for each system considering all replicates. Statistical significance was investigated via Student’s *t*-test utilizing Python SciPy library (version 1.11.3). [58] Python files and output files of the statistical tests were uploaded to the GitHub repository (https://github.com/ccbiozhaw/FAST-PETase_stability).

### 5.6 Hydrogen bonding analysis

All hydrogen bonds in which S121(E), D186(H), R224(Q), N233(K), N172, S192, S193, E204 and N233K are involved were detected using the MDTraj’s Baker-Hubbert hydrogen bonding analysis. [59] To identify the relevance of the resulting interactions, distances between all bonding atoms were calculated for all frames of all simulation systems. The interactions were ranked by the highest increase or decrease of their mean distances by comparing 30 °C and 50 °C systems.

### 5.7 Physical energy calculations

The output frames of all simulations described above were saved as PDB files which were used to analyze the impact of temperature on ΔG(Stability) for the different variants. For this purpose, stand-alone versions of the stability predictors EvoEF1 [31], EvoEF2 [32] and FoldX [33] were used. To enable batchwise analysis of multiple structures, Python scripts were developed (https://github.com/ccbiozhaw/FAST-PETase_stability). To ensure compatibility with the respective stability predictors, library-specific functions were applied to repair the PDB files. In this context, the term ‘repair’ refers to the optimization of incomplete side chains of a protein, whereby steric collisions are reduced as far as possible. The repaired PDB files were then used to calculate ΔG(Stability) using the respective stability predictor (Supplementary Table S3).

## CRediT authorship contribution statement

**Buller Rebecca:** Writing – review & editing, Writing – original draft, Supervision, Resources, Project administration, Funding acquisition, Formal analysis, Conceptualization. **Farnaud Sebastien:** Writing – review & editing, Writing – original draft, Supervision, Project administration, Conceptualization. **Niederhauser Cornel:** Writing – original draft, Visualization, Investigation. **Stockinger Peter:** Writing – review & editing, Writing – original draft, Visualization, Software, Methodology, Investigation, Formal analysis, Data curation, Conceptualization.

## Declaration of Competing Interest

The authors declare the following financial interests/personal relationships which may be considered as potential competing interests: Rebecca Buller reports financial support and article publishing charges were provided by Swiss National Science Foundation. If there are other authors, they declare that they have no known competing financial interests or personal relationships that could have appeared to influence the work reported in this paper.

## Footnotes

Appendix A Supplementary data associated with this article can be found in the online version at doi:10.1016/j.csbj.2025.03.006.
