# Supplementary material for: Computational analysis reveals temperature-induced stabilization of FAST-PETase
Source: Comput Struct Biotechnol J. 2025 Mar 5;27:969–77. doi: 10.1016/j.csbj.2025.03.006 (PMC11946493; doi:10.1016/j.csbj.2025.03.006)
Supplement: Supplementary file 1 — Supplementary material [file mmc1.pdf]

Supporting Information

Computational Analysis Reveals Temperature-Induced Stabilization of FAST-PETase

Authors

Peter Stockinger [1,2] (https://orcid.org/0000-0002-3494-7302),

Cornel Niederhauser [2] (https://orcid.org/0000-0002-8176-4250),

Sebastien Farnaud [1*] (https://orcid.org/0000-0003-2077-5797),

Rebecca Buller [2*] (https://orcid.org/0000-0002-5997-1616)

Affiliations

[1] Research Centre for Health & Life Sciences, Coventry University, CV1 5FB, Coventry, United Kingdom

[2] Competence Center for Biocatalysis, Zurich University of Applied Sciences, Einsiedlerstrasse 31, 8820 Wädenswil, Switzerland

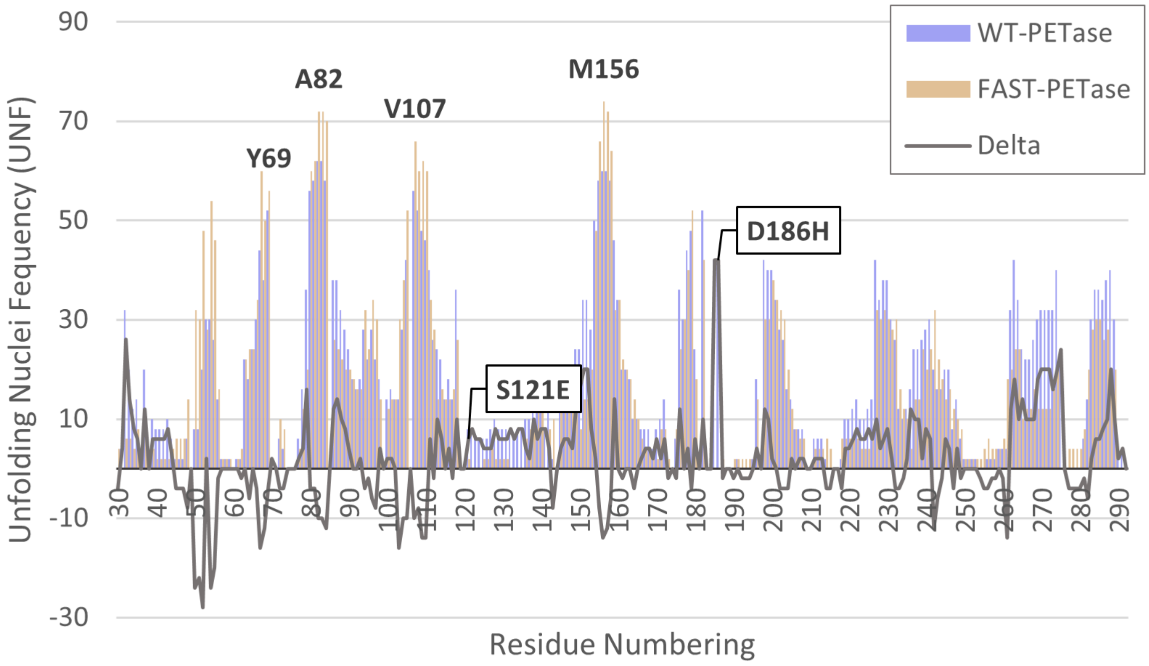

Fig. S1: CNAnalysis Identifies Unfolding Nuclei

Unfolding nuclei frequencies were calculated for WT- (blue) and FAST-PETase (orange). Residues around Y69, A82, V107 and M156 were identified to be potential hotspot position for further engineering as unfolding nuclei frequencies of WT- and FAST-PETase were found to be higher than 50. The solid line represents the delta between WT- and FAST-PETase frequencies (WT $PETase_{unfolding\ nuclei\ frequency}$ - FAST $PETase_{unfolding\ nuclei\ frequency}$). Positive delta values indicate the reduction of unfolding nuclei frequence of the respective residue in FAST-PETase, while negative values indicate a destabilizing effect of the introduced mutations. Positions of mutations S121E and D186H are highlighted with a box, as a notable drop in the unfolding nuclei frequency in FAST PETase compared to WT-PETase was observed.

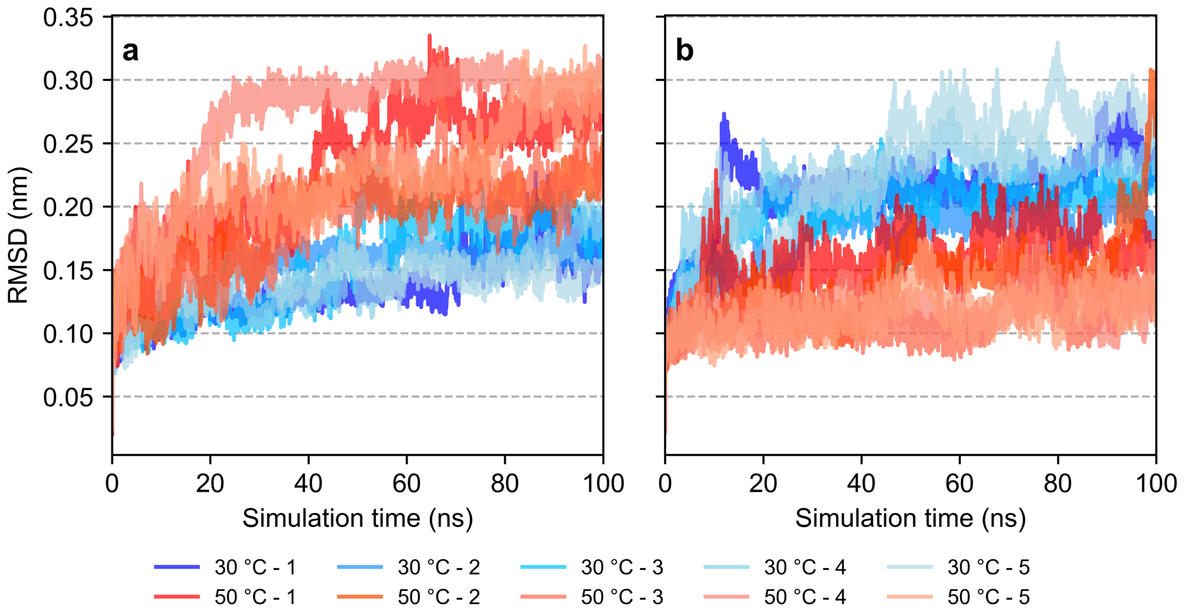

Fig. S2: MD Simulation-derived RMSD analysis

RMSD was calculated for all simulation replicates of simulations (**a**) WT- and (**b**) FAST-PETase. The 30 °C simulations are displayed using blue colors while the 50 °C simulations are displayed in shades of red. This Supplementary Figure shows the original data of Figure 2, to which we applied a Savitzky-Golay filter (window size: 11, polynomial order: 3) to reduce the noise. Original data was uploaded to the GitHub Repository (https://github.com/ccbiozhaw/FAST-PETase_stability).

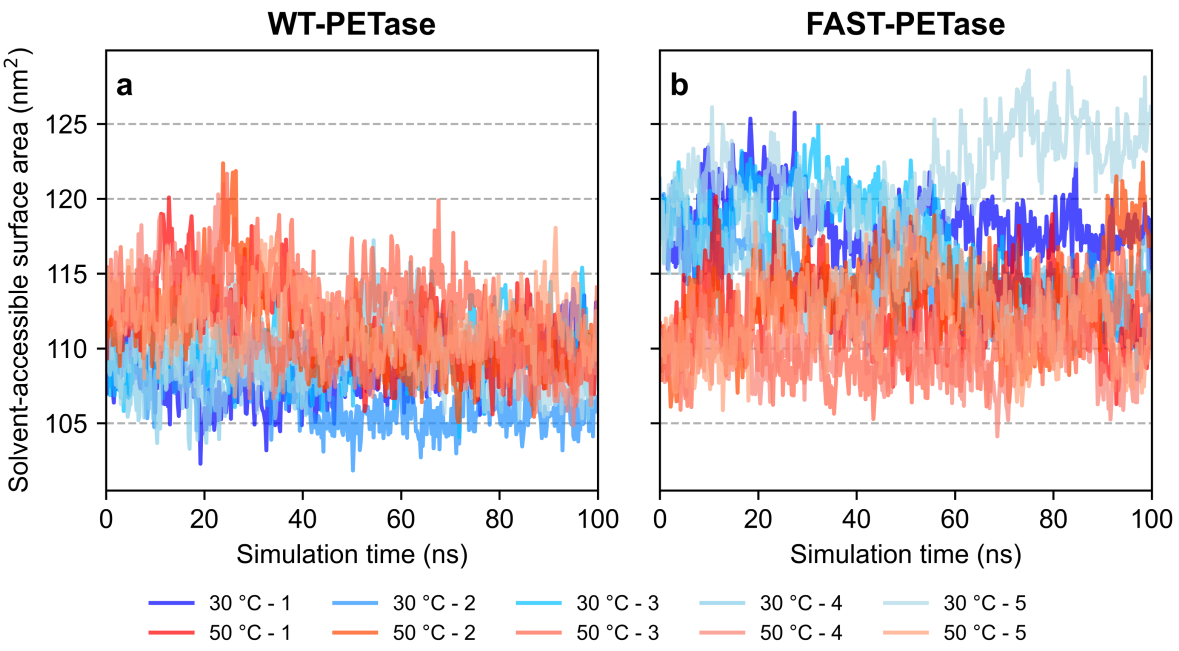

Fig. S3: MD Simulation-derived analysis of the solvent accessible surface area (SASA)

SASA was calculated after every 100 simulation steps for all simulation replicates of simulations (**a**) WT- and (**b**) FAST-PETase. The 30 °C simulations are displayed using blue colors while the 50 °C simulations are displayed in shades of red. Original data was uploaded to the GitHub Repository (https://github.com/ccbiozhaw/FAST-PETase_stability).

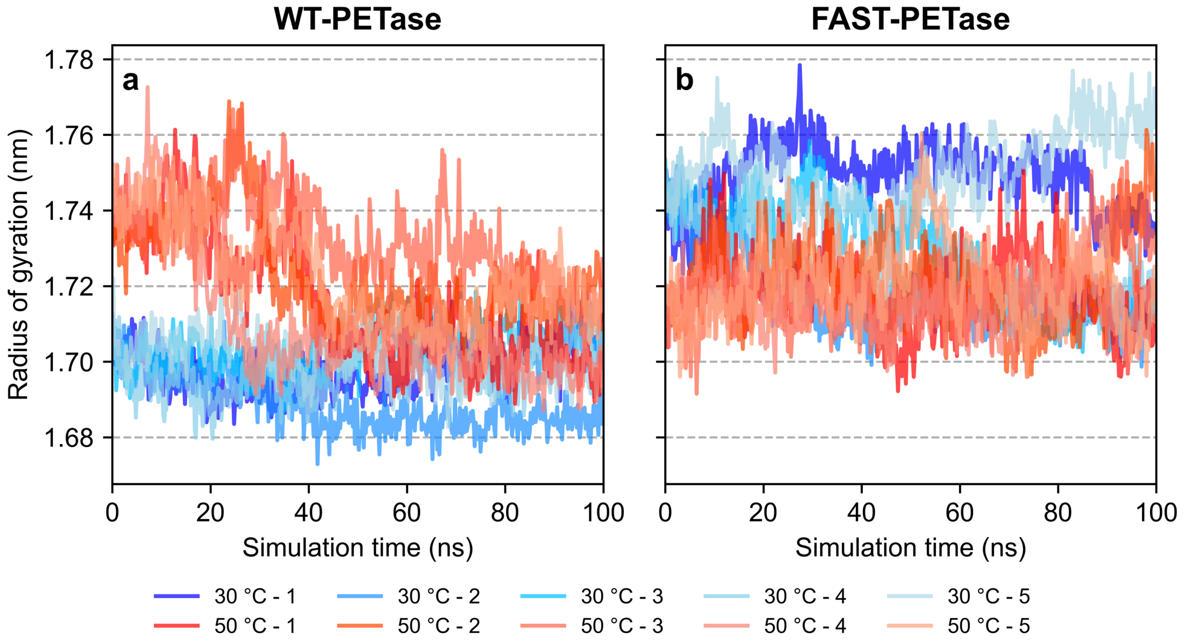

Fig. S4: MD Simulation-derived analysis of the radius of gyration (Rg)

Rg was calculated after every 100 simulation steps for all simulation replicates of simulations (**a**) WT- and (**b**) FAST-PETase. The 30 °C simulations are displayed using blue colors while the 50 °C simulations are displayed in shades of red. Original data was uploaded to the GitHub Repository (https://github.com/ccbiozhaw/FAST-PETase_stability).

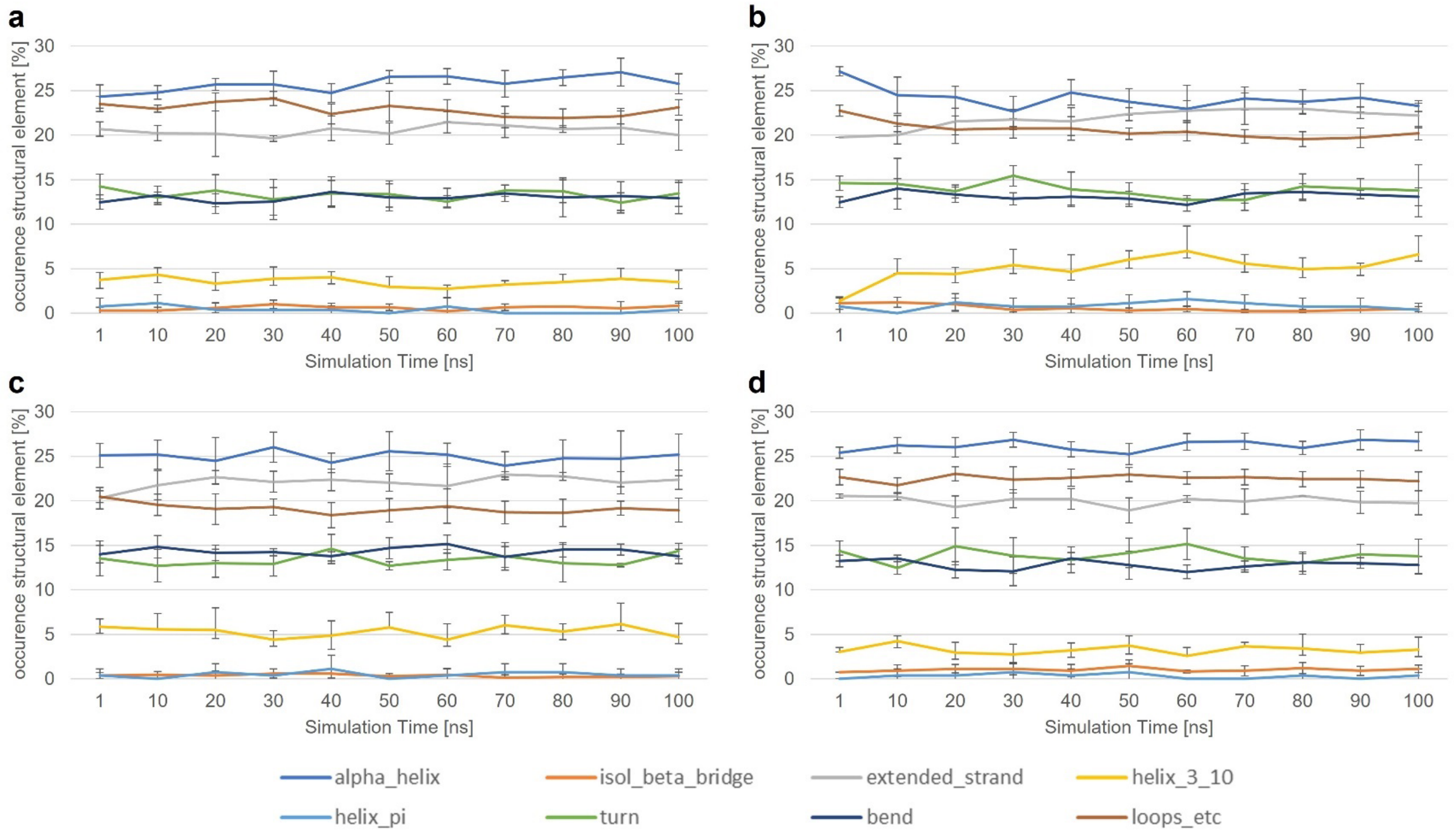

Fig. S5: MD Simulation-based Secondary Structure Analysis

Analysis of the mean occurrence of secondary structure elements (using MDTraj's implementation of the dictionary of protein secondary structure[1,2]) of selected frames (at 1, 10, 20, 30, 40, 50, 60, 70, 80, 90, and 100 ns) of the MD simulations carried out for WT-PETase at (a) 30°C and (b) 50°C and FAST-PETase at (c) 30°C and (d) 50 °C. Error bars represent the standard deviations calculated from all replicates.

Table S1: MD Simulation-based Hydrogen Bonding Analysis of WT-PETase

All atom pairs involved in hydrogen bonds detected for residues S121, D186, N172, S192, S193, E204, R224, and N233 were ranked by the difference (Δ) of their mean distance at 30 °C and 50 °C in all trajectories (Mean Distance $(nm)_{50\ °C}$ - Mean Distance $(nm)_{30\ °C}$). In this analysis, negative values indicate temperature-dependent bond strengthening while positive values correspond to bond weakening at the higher temperature.

Hydrogen Bond	Mean Distance [nm]		Δ [nm]
	30°C simulations	50°C simulations	
ASN233-ND2 -- ASP283-OD1	0.897	1.154	0.257
ASN233-ND2 -- ASP283-OD2	0.899	1.155	0.255
SER192-OG -- ASN190-OD1	0.357	0.567	0.210
SER192-N -- ASN190-OD1	0.362	0.568	0.206
SER187-OG -- ASP186-OD2	0.468	0.661	0.193
ASN190-ND2 -- SER192-OG	0.465	0.649	0.184
SER187-OG -- ASP186-OD1	0.485	0.663	0.178
GLU204-N -- GLY234-O	0.422	0.599	0.177
VAL211-N -- GLU204-OE2	0.823	0.991	0.168
VAL211-N -- GLU204-OE1	0.825	0.988	0.163
SER188-N -- ASP186-OD1	0.422	0.581	0.160
SER207-OG -- GLU204-O	0.735	0.888	0.153
SER188-N -- ASP186-OD2	0.429	0.579	0.150
ASN205-N -- GLY234-O	0.397	0.534	0.137
SER207-N -- GLU204-O	0.536	0.665	0.129
ASN233-ND2 -- GLU231-OE1	0.722	0.835	0.113
ASN172-ND2 -- ASN173-OD1	0.746	0.839	0.093
SER187-N -- ASP186-OD2	0.333	0.421	0.088
SER188-OG -- ASP186-OD1	0.602	0.690	0.088
GLN119-NE2 -- SER121-OG	0.579	0.664	0.085
SER187-N -- ASP186-OD1	0.344	0.422	0.078
SER188-N -- ASP186-O	0.317	0.394	0.077
ASN246-ND2 -- GLY234-O	0.628	0.697	0.069
SER188-OG -- ASP186-OD2	0.621	0.688	0.067
SER282-N -- ASN233-OD1	0.537	0.591	0.055
SER192-N -- SER221-OG	0.366	0.420	0.054
ASN172-ND2 -- ILE168-O	0.337	0.384	0.046
ASN233-ND2 -- ILE232-O	0.562	0.595	0.033
GLY165-N -- ASP186-OD1	0.707	0.740	0.033
ASP206-N -- GLU204-O	0.354	0.384	0.031
GLY165-N -- ASP186-OD2	0.705	0.734	0.028
ASN246-ND2 -- ASN233-O	0.547	0.570	0.023
ARG224-NE -- SER223-OG	0.561	0.583	0.023
SER192-OG -- SER221-O	0.429	0.450	0.021
ASN233-ND2 -- ARG280-O	0.538	0.557	0.019
VAL194-N -- SER192-O	0.346	0.362	0.016

Table S1 (continued): MD Simulation-based Hydrogen Bonding Analysis of WT-PETase

Hydrogen Bond	Mean Distance [nm] 30°C simulations	50°C simulations	Δ [nm]
ASN172-N -- SER169-O	0.335	0.351	0.016
ASN172-ND2 -- SER193-OG	0.829	0.842	0.013
THR195-OG1 -- ARG224-O	0.566	0.579	0.013
SER124-N -- SER121-O	0.347	0.359	0.012
SER188-OG -- ASP186-O	0.628	0.638	0.010
SER282-OG -- ASN233-OD1	0.537	0.546	0.009
ASN172-N -- ILE168-O	0.318	0.326	0.008
GLY234-N -- ILE232-O	0.351	0.359	0.008
THR195-OG1 -- SER193-O	0.476	0.483	0.007
ARG224-NH2 -- SER192-O	0.579	0.584	0.005
ARG224-NH1 -- SER192-O	0.528	0.533	0.005
ARG224-NE -- SER192-O	0.465	0.464	-0.001
SER122-N -- SER121-OG	0.351	0.350	-0.001
SER214-OG -- ASP186-O	0.570	0.568	-0.002
ARG280-NE -- ASN233-O	0.622	0.619	-0.003
ASN205-ND2 -- ASN233-O	0.733	0.729	-0.004
SER125-N -- SER121-O	0.318	0.314	-0.004
SER187-OG -- ASP186-O	0.488	0.483	-0.005
SER124-OG -- SER121-O	0.435	0.428	-0.007
ARG224-N -- SER192-O	0.497	0.489	-0.007
ASN205-ND2 -- GLY234-O	0.474	0.459	-0.015
ARG280-NE -- ASN233-OD1	0.763	0.747	-0.017
SER236-N -- GLY234-O	0.394	0.376	-0.018
ASP186-N -- SER214-OG	0.542	0.522	-0.020
SER125-OG -- SER121-O	0.444	0.418	-0.026
GLY234-N -- ARG280-O	0.625	0.594	-0.031
GLU204-N -- ILE232-O	0.344	0.310	-0.034
GLY235-N -- ASN233-O	0.348	0.313	-0.034
ARG224-NH2 -- SER193-O	0.691	0.654	-0.037
ASP186-N -- ASP186-OD2	0.449	0.411	-0.038
ARG224-NH1 -- SER193-O	0.606	0.567	-0.039
ARG224-NE -- SER193-O	0.591	0.547	-0.044
VAL211-N -- GLU204-O	0.638	0.591	-0.047
SER193-OG -- ASN190-OD1	0.674	0.627	-0.047
ASP186-N -- ASP186-OD1	0.455	0.406	-0.049
ARG280-NH1 -- ASN233-O	0.637	0.584	-0.053
ARG280-NH1 -- ASN233-OD1	0.785	0.728	-0.057
ARG280-NH2 -- ASN233-OD1	0.864	0.806	-0.058
ARG280-NH2 -- ASN233-O	0.718	0.659	-0.059
ASP186-N -- PRO184-O	0.482	0.423	-0.059
ASN172-ND2 -- SER193-O	0.983	0.922	-0.060
SER192-N -- ASN190-O	0.421	0.359	-0.062
ASN233-N -- ARG280-O	0.355	0.293	-0.063
ARG280-NH1 -- GLU204-OE1	1.187	1.119	-0.068
ASN233-ND2 -- VAL281-O	0.630	0.560	-0.070

Table S1 (continued): MD Simulation-based Hydrogen Bonding Analysis of WT-PETase

Hydrogen Bond	Mean Distance [nm]		Δ [nm]
	30°C simulations	50°C simulations	
**ASP186-N -- GLY163-O**	0.743	0.673	-0.071
**ARG280-NH2 -- GLU204-OE1**	1.270	1.196	-0.074
**ARG280-NH2 -- GLY234-O**	0.968	0.892	-0.077
**ARG280-NH1 -- GLU204-OE2**	1.190	1.112	-0.078
**ASN233-ND2 -- SER282-OG**	0.544	0.462	-0.082
**ASN233-ND2 -- THR279-O**	0.553	0.470	-0.083
**ARG280-NH2 -- GLU204-OE2**	1.273	1.188	-0.085
**TRP185-N -- ASP186-OD2**	0.749	0.635	-0.114
**ASN172-ND2 -- THR195-OG1**	1.173	1.054	-0.119
**GLY234-N -- GLU204-OE1**	0.522	0.400	-0.122
**ASN244-ND2 -- GLY234-O**	1.042	0.911	-0.131
**THR195-OG1 -- ASN172-OD1**	1.144	1.011	-0.133
**GLY234-N -- GLU204-OE2**	0.526	0.393	-0.133
**SER192-N -- THR189-O**	0.577	0.436	-0.141
**GLY164-N -- ASP186-OD1**	0.758	0.614	-0.145
**GLY164-N -- ASP186-OD2**	0.757	0.608	-0.149
**ASN233-ND2 -- GLU204-OE1**	0.622	0.466	-0.157
**ASN233-ND2 -- GLU204-OE2**	0.625	0.461	-0.164
**SER193-OG -- ASN190-O**	0.594	0.424	-0.170
**SER193-N -- ASN190-O**	0.586	0.416	-0.170
**GLY162-N -- ASP186-OD2**	0.979	0.788	-0.191
**GLY162-N -- ASP186-OD1**	0.990	0.784	-0.205
**ASP186-N -- GLY162-O**	0.932	0.703	-0.230
**GLY164-N -- ASP186-O**	0.838	0.608	-0.230
**SER192-OG -- THR189-O**	0.756	0.480	-0.275
**GLY163-N -- ASP186-OD2**	1.007	0.675	-0.332
**GLY163-N -- ASP186-OD1**	1.011	0.673	-0.338
**SER282-OG -- GLU204-OE1**	0.790	0.410	-0.381
**SER282-OG -- GLU204-OE2**	0.791	0.410	-0.381

Table S2: MD Simulation-based Hydrogen Bonding Analysis of FAST-PETase

All atom pairs involved in hydrogen bonds detected for E121, H186, N172, S192, S193, E204, Q224, and K233 were ranked by the difference (Δ) of their mean distance at 30 °C and 50 °C in all trajectories (Mean Distance $(nm)_{50\ °C}$ - Mean Distance $(nm)_{30\ °C}$). In this analysis, negative values indicate temperature-dependent bond strengthening while positive values correspond to bond weakening at the higher temperature.

Hydrogen Bond	Mean Distance [nm]		Δ [nm]
	30°C simulations	50°C simulations	
SER193-OG -- HIS186-ND1	0.525	1.292	0.766
HIS186-NE2 -- SER193-OG	0.411	1.161	0.750
TRP185-NE1 -- GLU121-OE2	0.715	1.451	0.736
TRP185-NE1 -- GLU121-OE1	0.717	1.451	0.734
SER188-OG -- SER193-OG	0.417	1.073	0.656
ASN288-ND2 -- GLN224-OE1	0.547	0.974	0.427
GLU204-N -- GLY234-O	0.314	0.713	0.398
HIS186-NE2 -- LEU167-O	0.503	0.859	0.356
ASN288-ND2 -- GLN224-O	0.355	0.680	0.325
SER193-OG -- ASN190-O	0.322	0.616	0.293
GLY235-N -- GLU204-OE1	0.642	0.919	0.278
SER193-N -- ASN190-O	0.338	0.603	0.265
GLY235-N -- GLU204-OE2	0.640	0.899	0.259
HIS186-NE2 -- ASN190-O	0.516	0.721	0.205
GLY234-N -- GLU204-OE1	0.483	0.686	0.203
GLY234-N -- GLU204-OE2	0.481	0.671	0.190
SER207-OG -- GLU204-O	0.690	0.848	0.158
SER223-N -- SER192-OG	0.493	0.637	0.144
GLU121-N -- GLN119-OE1	0.488	0.626	0.138
ASN205-ND2 -- GLU204-OE1	0.523	0.659	0.136
SER207-N -- GLU204-O	0.499	0.635	0.135
GLN119-NE2 -- GLU121-OE2	0.630	0.763	0.133
GLN119-NE2 -- GLU121-OE1	0.633	0.766	0.133
ASN205-N -- GLY234-O	0.477	0.599	0.122
ASN205-ND2 -- GLU204-OE2	0.519	0.641	0.122
VAL211-N -- GLU204-OE1	0.812	0.927	0.115
GLY235-N -- LYS233-O	0.388	0.503	0.115
ARG132-NH2 -- ASN172-OD1	1.151	1.264	0.113
LYS233-NZ -- THR279-O	0.388	0.495	0.107
SER192-N -- ASN190-O	0.330	0.435	0.105
SER193-OG -- ASN190-OD1	0.585	0.664	0.079
GLY234-N -- ILE232-O	0.340	0.415	0.075
SER238-OG -- GLY234-O	0.726	0.794	0.068
ASN73-ND2 -- GLU204-O	4.280	4.344	0.064
SER193-N -- ASN190-OD1	0.520	0.564	0.045
SER124-OG -- GLU121-O	0.408	0.445	0.037

Table S2 (continued): MD Simulation-based Hydrogen Bonding Analysis of FAST-PETase

Hydrogen Bond	Mean Distance [nm]		Δ [nm]
	30°C simulations	50°C simulations	
GLN224-N -- SER223-OG	0.328	0.359	0.031
GLN224-N -- GLN224-OE1	0.440	0.470	0.030
SER192-N -- SER221-O	0.367	0.397	0.029
ASN172-N -- SER169-O	0.317	0.341	0.025
SER122-OG -- GLU121-OE1	0.795	0.805	0.010
SER122-OG -- GLU121-OE2	0.792	0.801	0.009
LYS233-N -- ALA280-O	0.301	0.309	0.008
SER187-OG -- GLU121-OE1	0.850	0.857	0.008
SER192-OG -- SER221-O	0.420	0.423	0.004
GLN224-NE2 -- GLN224-O	0.579	0.582	0.003
SER187-N -- HIS186-ND1	0.313	0.312	-0.001
SER193-OG -- SER193-O	0.374	0.371	-0.003
SER188-N -- HIS186-O	0.355	0.352	-0.003
SER122-N -- GLU121-OE1	0.543	0.539	-0.004
SER188-OG -- HIS186-ND1	0.600	0.595	-0.005
SER236-N -- GLY234-O	0.449	0.442	-0.007
SER122-N -- GLU121-OE2	0.544	0.538	-0.007
SER187-OG -- GLU121-OE2	0.865	0.856	-0.009
SER188-N -- HIS186-ND1	0.407	0.397	-0.010
ASN190-ND2 -- SER192-OG	0.504	0.493	-0.011
SER124-N -- GLU121-O	0.361	0.349	-0.012
THR195-N -- SER193-O	0.366	0.348	-0.018
GLU204-N -- ALA202-O	0.431	0.408	-0.024
GLU121-N -- GLU121-OE2	0.458	0.434	-0.024
ASP206-N -- GLU204-O	0.384	0.359	-0.025
SER223-OG -- SER192-OG	0.613	0.586	-0.027
SER192-OG -- SER223-OG	0.613	0.586	-0.027
THR195-OG1 -- SER193-O	0.519	0.483	-0.036
SER192-OG -- THR189-O	0.785	0.749	-0.037
ASN172-ND2 -- ILE168-O	0.397	0.355	-0.042
ASN172-N -- ILE168-O	0.356	0.306	-0.050
SER192-N -- THR189-O	0.633	0.580	-0.053
SER192-OG -- ASN190-OD1	0.448	0.389	-0.059
SER192-N -- ASN190-OD1	0.445	0.383	-0.062
SER125-N -- GLU121-O	0.373	0.310	-0.064
SER125-OG -- GLU121-O	0.495	0.429	-0.066
HIS186-NE2 -- SER188-O	0.543	0.470	-0.073
GLY234-N -- ASN246-OD1	0.735	0.653	-0.082
GLY234-N -- ASN205-OD1	0.727	0.634	-0.093
GLU204-N -- ILE232-O	0.422	0.326	-0.096
ARG53-NH2 -- GLU204-O	4.477	4.377	-0.101
ASN172-ND2 -- ASN173-OD1	0.907	0.794	-0.114
LYS233-NZ -- SER282-OG	0.630	0.498	-0.132
HIS237-N -- GLY234-O	0.777	0.639	-0.138
ASN205-ND2 -- GLY234-O	0.596	0.451	-0.144

Table S2 (continued): MD Simulation-based Hydrogen Bonding Analysis of FAST-PETase

Hydrogen Bond	Mean Distance [nm]		Δ [nm]
	30°C simulations	50°C simulations	
LYS233-NZ -- GLU231-OE1	0.966	0.820	-0.146
ARG53-NH1 -- GLN224-O	4.136	3.985	-0.152
LYS233-NZ -- GLU231-OE2	0.969	0.817	-0.152
GLN224-NE2 -- SER223-OG	0.711	0.559	-0.152
ARG53-NH2 -- GLU204-OE1	4.654	4.502	-0.152
SER193-OG -- ILE168-O	0.951	0.795	-0.156
ARG53-NH2 -- GLU204-OE2	4.654	4.496	-0.157
ARG53-NH1 -- GLU204-OE1	4.619	4.461	-0.158
ARG53-NH1 -- GLU204-OE2	4.619	4.456	-0.163
THR56-N -- SER192-OG	4.025	3.847	-0.178
THR56-OG1 -- SER192-OG	4.103	3.915	-0.189
ASN244-ND2 -- GLY234-O	0.970	0.777	-0.193
SER282-OG -- GLU204-OE2	0.775	0.575	-0.199
SER282-OG -- GLU204-OE1	0.773	0.557	-0.216
SER192-N -- SER221-OG	0.576	0.357	-0.219
ARG53-NH2 -- GLN224-OE1	4.640	4.406	-0.234
ARG53-NE -- GLN224-OE1	4.487	4.249	-0.238
ARG53-NH1 -- GLN224-OE1	4.561	4.322	-0.239
ASN246-ND2 -- LYS233-O	0.608	0.353	-0.255
GLY165-N -- HIS186-NE2	0.942	0.676	-0.266
SER54-N -- GLN224-OE1	4.126	3.839	-0.287
ARG90-NH1 -- GLN224-OE1	4.184	3.895	-0.289
ASN244-ND2 -- LYS233-O	1.017	0.681	-0.336
GLN224-NE2 -- TYR70-OH	4.293	3.956	-0.337
GLN224-NE2 -- SER54-O	4.374	3.993	-0.381
LYS233-NZ -- GLU204-OE2	1.036	0.636	-0.400
LYS233-NZ -- GLU204-OE1	1.036	0.632	-0.404
GLN224-NE2 -- VAL194-O	1.130	0.642	-0.488
GLN224-NE2 -- SER192-O	0.990	0.482	-0.508
HIS186-NE2 -- GLU121-OE1	1.178	0.657	-0.521
HIS186-NE2 -- GLU121-OE2	1.190	0.658	-0.532
ARG59-NH2 -- GLN224-OE1	4.724	4.168	-0.556
THR195-OG1 -- GLN224-O	1.158	0.599	-0.559
ARG59-NE -- GLN224-OE1	4.639	4.075	-0.564
GLN224-NE2 -- GLY64-O	4.218	3.635	-0.582
GLN224-NE2 -- SER58-O	4.626	4.023	-0.603
GLN224-NE2 -- THR195-OG1	1.449	0.787	-0.662
HIS186-N -- SER214-OG	1.258	0.386	-0.872
SER214-OG -- HIS186-O	1.395	0.373	-1.022

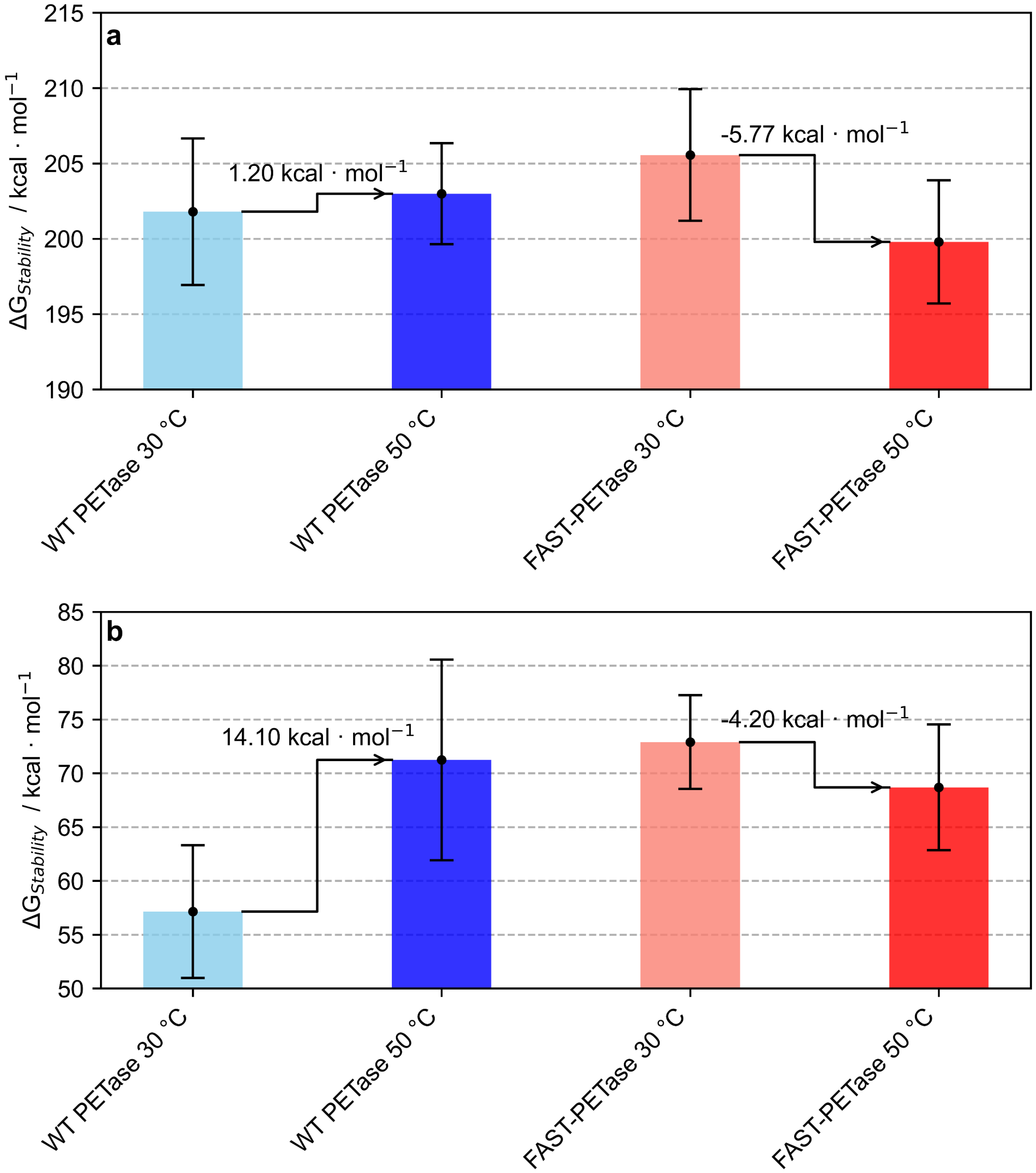

Fig. S6: MD Simulations-derived Physical Energy Calculations

Comparison of the average (a) EvoEF1 and (b) FoldX ΔG(Stability) values derived using the five end frames from the MD simulations of WT- and FAST-PETase (blue and red, respectively). The lighter colors correspond to calculation based on the MD simulations at 30°C and the darker colors show the values derived from the MD simulations at 50°C. The error bars show the standard deviation of the five end frames. In contrast to EvoEF2 (Fig. 5), the here-applied energy functions resulted in overall positive ΔG values, however, the derived ΔΔG values show the same trend as derived from EvoEF2 calculations.

Table S3: MD Simulations-derived ΔG(Stability) Values

ΔG(Stability) values derived from the five end frames from the MD simulations of WT- and FAST-PETase at 30 °C and 50 °C calculated with and FoldX[3],. EvoEF1[4], and EvoEF2[5].

		ΔG(Stability) / kcal · mol^{-1}		
**Variant**	**Replica**	**FoldX**	**EvoEF1**	**EvoEF2**
Wildtype 30 °C	1	61.78	201.28	-1079.79
	2	47.69	193.68	-1126.85
	3	65.4	203.68	-1080.47
	4	53.82	206.46	-1070.47
	5	57.02	203.87	-1104.26
Wildtype 50 °C	1	55.47	206.63	-1062.02
	2	83.7	198.71	-1037.24
	3	75.15	198.76	-1054.18
	4	68.2	208.77	-1025.86
	5	73.68	202.08	-1065.23
FAST-PETase 30 °C	1	71.95	202.86	-1074.84
	2	80.7	201.67	-1060.6
	3	69.53	200.68	-1095.82
	4	68.34	209.53	-1067.96
	5	73.95	213.07	-1021.49
FAST-PETase 50 °C	1	70.25	202.37	-1092.44
	2	75.85	203.67	-1079.01
	3	63.1	201.3	-1106.83
	4	73.5	196.43	-1129.67
	5	60.77	195.21	-1138.43

1. McGibbon, R. T. *et al.* MDTraj: A Modern Open Library for the Analysis of Molecular Dynamics Trajectories. *Biophys J* (2015) doi:10.1016/j.bpj.2015.08.015.
2. Kabsch, W. & Sander, C. Dictionary of protein secondary structure: pattern recognition of hydrogen-bonded and geometrical features. *Biopolymers* **22**, 2577–2637 (1983).
3. Delgado, J., Radusky, L. G., Cianferoni, D. & Serrano, L. FoldX 5.0: Working with RNA, small molecules and a new graphical interface. *Bioinformatics* (2019) doi:10.1093/bioinformatics/btz184.
4. Pearce, R., Huang, X., Setiawan, D. & Zhang, Y. EvoDesign: Designing Protein–Protein Binding Interactions Using Evolutionary Interface Profiles in Conjunction with an Optimized Physical Energy Function. *J Mol Biol* (2019) doi:10.1016/j.jmb.2019.02.028.
5. Huang, X., Pearce, R. & Zhang, Y. EvoEF2: Accurate and fast energy function for computational protein design. *Bioinformatics* (2020) doi:10.1093/bioinformatics/btz740.
